# Mistreatment and Discrimination during Childbirth, Associations with Symptoms of Childbirth-Related Posttraumatic Stress Disorder and the Mediating Role of the Subjective Birth Experience: A Quantitative Analysis Within the Prospective Cohort Study RESPECT _PARENTS_

**DOI:** 10.1055/a-2717-7798

**Published:** 2025-11-11

**Authors:** Christin Porstendorfer-Almeida Froz, Bianka Vollert, Ionna Hansen, Nina Schurig, Lara Seefeld, Victoria Weise, Cahit Birdir, Pauline Wimberger, Susan Garthus-Niegel

**Affiliations:** 1 59988 Institute and Policlinic of Occupational and Social Medicine, TUD Dresden University of Technology, Dresden, Germany; 2 686782 Institute for Systems Medicine (ISM), Faculty of Medicine, MSH Medical School Hamburg, Hamburg, Germany; 3 210417 Institute for Medical Information Processing, Biometry, and Epidemiology, Pettenkofer School of Public Health, Ludwig-Maximilians-University Munich, Munich, Germany; 4 3688 Department of Psychology & Neuroscience, Dalhousie University, Halifax, Canada; 5 59988 Department of Child and Adolescent Psychiatry, Faculty of Medicine and University Hospital Carl Gustav Carus, TUD Dresden University of Technology, Dresden, Germany; 6 59988 Department of Gynecology and Obstetrics, Faculty of Medicine and University Hospital Carl Gustav Carus, TUD Dresden University of Technology, Dresden, Germany; 7 152528 Department of Obstetrics and Gynecology, University of Heidelberg, Heidelberg, Germany; 8 25563 Department of Childhood and Families, Norwegian Institute of Public Health, Oslo, Norway

**Keywords:** RESPECT study, obstetric violence/mistreatment, discrimination, subjective birth experience, CB-PTSD symptoms, RESPECT-Studie, Gewalt in der Geburtshilfe, Diskriminierung, subjektives Geburtserleben, geburtsbezogene PTBS-Symptome

## Abstract

**Background:**

Mistreatment and discrimination during childbirth are a global public health concern. Such experiences can adversely affect the subjective birth experience and increase the likelihood of compromised postpartum mental health, including symptoms of childbirth-related posttraumatic stress disorder (CB-PTSD) not only in mothers/birthing parents, but also in partners. This study examines instances of mistreatment and discrimination during childbirth, their association with CB-PTSD symptoms, and the potential mediating role of the subjective birth experience in both parents.

**Methods:**

Data were retrieved from the prospective RESPECT _PARENTS_ cohort, a German community sample. For the current study, data from n = 1075 mothers/birthing parents and n = 454 partners, collected at two assessment points, i.e., during pregnancy and eight weeks after birth, were included. Regression and mediation models were used to analyze the associations separately for mothers/birthing parents and partners.

**Results:**

Mistreatment during childbirth was reported by 25.8% of mothers/birthing parents and 6.8% of partners, whereas perceived discrimination was reported by 3.9% of mothers/birthing parents and 3.5% of partners. These experiences predicted more severe CB-PTSD symptoms among mothers/birthing parents. For both parents, experiencing more mistreatment and more discrimination was associated with a more negative subjective birth experience, which in turn was associated with more severe CB-PTSD symptoms, demonstrating a significant mediation effect.

**Conclusion:**

The current study indicates that mistreatment and discrimination during childbirth exist in Germany among both mothers/birthing parents and partners, potentially contributing to a more negative subjective birth experience and more severe CB-PTSD symptoms. The findings emphasize the need to address mistreatment and discrimination during childbirth for both parents, in general and as potential risk factors for adverse outcomes.

## Zusammenfassung

**Hintergrund:**

Gewalt und Diskriminierung unter der Geburt stellen ein weltweites Problem der öffentlichen Gesundheit dar. Derartige Erfahrungen können das subjektive Geburtserleben beeinträchtigen und die Wahrscheinlichkeit von psychischen Problemen nach der Geburt, einschließlich Symptomen einer geburtsbezogenen Posttraumatischen Belastungsstörung (PTBS), sowohl für Mütter/Gebärende als auch Partner*innen erhöhen. Diese Studie untersucht das Auftreten von Gewalt und Diskriminierung unter der Geburt, den Zusammenhang mit geburtsbezogenen PTBS-Symptomen sowie die potenziell vermittelnde Rolle des subjektiven Geburtserlebens für beide Elternteile.

**Methoden:**

Die Daten stammen aus der prospektiven Kohorte RESPECT _PARENTS_ , einer in Deutschland rekrutierten bevölkerungsbasierten Stichprobe. Für die aktuelle Studie wurden Daten von n = 1075 Müttern/Gebärenden und n = 454 Partner*innen ausgewertet, die zu 2 Messzeitpunkten (während der Schwangerschaft und 8 Wochen nach der Geburt) erhoben wurden. Es wurden Regressions- und Mediationsmodelle verwendet, um die Zusammenhänge für Mütter/Gebärende und Partner*innen in separaten Analysen zu betrachten.

**Ergebnisse:**

Gewalterfahrungen unter der Geburt wurden von 25,8% der Mütter/Gebärenden und 6,8% der Partner*innen berichtet, während Diskriminierung von 3,9% der Mütter/Gebärenden und 3,5% der Partner*innen berichtet wurde. Diese Erfahrungen sagten schwerwiegendere geburtsbezogene PTBS-Symptome bei Müttern/Gebärenden vorher. Für beide Elternteile war das Erleben von mehr Gewalt und Diskriminierung mit einem negativeren subjektiven Geburtserleben verbunden, welches wiederum mit schwerwiegenderen geburtsbezogenen PTBS-Symptomen assoziiert war und einen signifikanten Mediationseffekt zeigte.

**Schlussfolgerungen:**

Die vorliegende Studie zeigt, dass Gewalt und Diskriminierung unter der Geburt in Deutschland sowohl bei Müttern/Gebärenden als auch bei Partner*innen auftreten und zu einem negativerem Geburtserleben und schwerwiegenderen geburtsbezogenen PTBS-Symptomen beitragen können. Die Ergebnisse unterstreichen die Notwendigkeit, Gewalt und Diskriminierung beider Elternteile unter der Geburt sowohl allgemein als auch als potenzielle Risikofaktoren für negative Folgen zu adressieren.

## Introduction

Globally, reports of obstetric maternal mistreatment during childbirth give rise to concerns regarding the standards of obstetric health care 1 . The World Health Organization (WHO) has reaffirmed its commitment to the implementation of evidence-based obstetric health care with its 2018 recommendation 2 . In addition to the longstanding goal of reducing morbidity and mortality of mothers and infants, the recommendation now also includes the aim to provide respectful care in order to improve the subjective birth experience for mothers and thereby laying the foundation for healthy and positive physical and mental development after childbirth for both mothers and their families.

Accordingly, there has been a shift toward acknowledging that expectant mothers/birthing parents may encounter behaviors during childbirth that violate their right to respectful obstetric health care and (bodily) autonomy 2 . These behaviors, whether intentional or not, can manifest at personal (i.e., individual actions of healthcare providers), institutional (i.e., policies within healthcare facilities), and structural levels (i.e., overarching societal systems) within obstetric health care 3 4 5 . Institutional protocols that fail to center the needs and dignity of mothers/birthing parents can facilitate environments where mistreatment during childbirth is normalized 6 . The power dynamics within health care settings, shaped by organizational structures and hierarchical systems, may intensify these challenges by creating conditions that allow mistreatment during childbirth to persist. This is often facilitated by institutional practices and limited accountability mechanisms that enable such behaviors to continue 7 . Given the complexity of these dynamics, a number of intersectional terms, including “mistreatment” 1 , “obstetric violence” 8 , “abuse”, and “disrespectful care” 9 have emerged to describe these experiences. To date, there is no consensus in the literature regarding a definition of mistreatment during childbirth 10 , but in a frequently cited publication by Freedman et al. (2024), mistreatment during childbirth is defined as “interactions or facility conditions that local consensus deems to be humiliating or undignified, and those interactions or conditions that are experienced as or intended to be humiliating or undignified” 4 . Discrimination represents a specific form of mistreatment that both precipitates and perpetuates mistreatment within obstetric health care contexts 6 . It is a particularly significant global issue that extends beyond obstetric health care, contributing to mistreatment across all areas of the health care system 11 .

The nature of mistreatment during childbirth can vary widely. Collectively, these terms describe behaviors of obstetric health care staff, or systemic conditions in facilities, that are perceived or experienced as humiliating or degrading 4 . In 2015, Bohren and colleagues introduced a typology for classifying mistreatment during childbirth, delineating seven categories: physical abuse, verbal abuse, sexual abuse, stigma and discrimination, failure to meet professional standards of care, poor rapport between women and providers, and health system conditions 1 . As defined in this typology, discrimination constitutes a form of mistreatment and is therefore included in the term. Generally, discrimination during childbirth refers to unequal, less favorable, or disrespectful treatment of (expectant) parents on the basis of specific characteristics such as ethnicity, age, religion, socioeconomic status, disabilities, or other (protected) attributes, leading to denial of equal and respectful care 12 . In addition, diverging opinions between parents and obstetric health care staff regarding appropriate medical interventions during childbirth have previously been considered a possible cause of poor treatment and perceived discrimination 13 .

A few studies have highlighted that non-dignified care, characterized by negative and disrespectful attitudes of obstetric health care providers, emerged as the most commonly reported form of mistreatment during childbirth 1 14 15 16 . Physical abuse in obstetric health care facilities was less frequently reported but remained a significant issue 16 .

Research has long highlighted deficiencies in obstetric health care in low- and middle-income countries 16 17 18 . Nevertheless, despite the general paucity of research, mistreatment during childbirth is also reported in high-income countries such as Germany 19 , the Netherlands 20 21 , France 22 , and the United States 6 23 . There is considerable variation in prevalence rates 3 , and comparisons are difficult due to substantial differences in study design and sample composition. In a non-representative German study, 77.6% of 2045 mothers who gave birth between 2009 and 2018 reported at least one instance of mistreatment during childbirth 19 . However, this high prevalence must be interpreted in light of a selection bias due to exclusive recruitment via social media and the overall aim of the study to validate an instrument for assessing experiences of disrespectful and abusive treatment of women during childbirth 19 . Two studies conducted in the United States found that 13.4% 6 and 17.3% 23 of mothers/birthing parents, respectively, reported at least one instance of mistreatment during childbirth, with the first study utilizing a representative sample and the second study employing a more diverse sample.

The variability in reported prevalence rates and the paucity of systematic research investigating mistreatment during childbirth in high-income countries complicates addressing the issue 24 . Additionally, the majority of studies focuses on the experiences of mothers/birthing parents, with minimal attention directed towards the experiences of partners. However, it is important to note that partners may also experience mistreatment during childbirth, such as exclusion, lack of information, or disrespectful treatment by obstetric health care staff 25 26 . Therefore, the experiences of mistreatment can adversely affect both mothers/birthing parents and partners 20 27 28 29 .

The consequences of mistreatment during childbirth are manifold for parents. As certain forms of obstetric mistreatment relate to an increased number of medical interventions during childbirth (e.g., physical abuse, failure to meet professional standards of care, non-consented interventions), potential consequences for mothers/birthing parents may include physical injury or associated outcomes like increased blood loss and additional pain 30 . Such experiences can prevent a natural childbirth experience and undermine women’s autonomy by limiting their control over the process and imposing medical procedures that may not align with their preferences or needs 31 . In addition, there can be serious consequences for the short- and long-term mental health of families, including the development of postpartum depression or postpartum anxiety disorders 32 33 . Additionally, there is a growing body of research focusing on childbirth-related posttraumatic stress disorder (CB-PTSD) 34 . It affects mothers/birthing parents following a traumatic childbirth with a prevalence of 4.7% and partners with a prevalence of 1.2% in community samples 35 . In high-risk samples, an average of 6.8% of mothers/birthing parents are affected, while no differences between community and high-risk samples regarding prevalence rates among partners were found 35 . Studies have shown significant associations between CB-PTSD and impaired parent-child bonding, couple relationship satisfaction, self-esteem, and future family planning 36 37 38 39 . Furthermore, CB-PTSD may result in significant financial impact, including decreased work productivity and costs associated with outpatient therapy and inpatient care for health care systems and affected families 40 41 . In the light of the numerous described adverse consequences for families and societies, CB-PTSD needs to be prevented wherever possible.

To date, there has been limited research, particularly in high-income countries, to determine how often parents are mistreated or discriminated during childbirth and whether and to what extent experiences of mistreatment and discrimination are associated with the development and severity of CB-PTSD symptoms. Furthermore, the role of the subjective birth experience in this context remains undetermined. Importantly, not all parents who experience mistreatment during childbirth subsequently develop CB‑PTSD 42 . The subjective birth experience, which is shaped by a variety of factors including obstetric health care, medical events during childbirth, and individual factors, such as mental health prior to or during pregnancy, and social support 43 44 , has been associated with the development of CB-PTSD 45 46 47 . It could play a crucial role in this context, as it can affect the interpretation and emotional response to events during childbirth. This in turn may determine the likelihood of developing CB-PTSD and may therefore act as a mediating factor in the association between mistreatment and/or discrimination and the development of subsequent CB-PTSD 27 .

Therefore, this study aims to 1) assess the prevalence of mistreatment and discrimination during childbirth for mothers/birthing parents and partners in a German sample, 2) explore the associations between mistreatment and discrimination during childbirth, the subjective birth experience, and CB-PTSD symptoms at eight weeks postpartum, and 3) test whether the association between mistreatment and CB-PTSD symptoms as well as the association between discrimination and CB-PTSD symptoms is mediated by the subjective birth experience.

The hypotheses are as follows:

1. The more instances of mistreatment and discrimination experienced during childbirth, the more severe the CB-PTSD symptoms will be at eight weeks postpartum.
2. The more instances of mistreatment and discrimination experienced during childbirth, the more negative the subjective birth experience will be at eight weeks after birth.
3. The more negative the subjective birth experience is, the more severe the CB-PTSD symptoms will be eight weeks after birth.
4. The association between mistreatment and discrimination during childbirth and CB-PTSD symptoms is mediated by the subjective birth experience.

## Methods

### Design

The current study is based on data from the interdisciplinary research project RESPECT (“A Prospective Mixed-Methods **RE** search Project on **S** ubjective Birth Experience and **PE** rson‑Centered **C** are in Paren **T** s and Obstetric Health Care Staff”) that is funded by the German Federal Ministry of Health. The overarching objective of RESPECT is to systematically elucidate the subjective birth experience of parents, with the long-term aim of improving obstetric health care practices to better align with parents’ needs. Within the ongoing quantitative main study RESPECT _PARENTS_ , the focus lies on investigating the subjective birth experiences of both mothers/birthing parents and partners, alongside associated factors before, during, and after childbirth. Data are collected at four assessment points: T1 during late pregnancy (at least 24 ^th^ gestational week), T2 at eight weeks after the anticipated birth date, T3 at six months after the actual birth date, and T4 at 24 months after the actual birth date. T2 consists of a structured telephone interview, while all other questionnaires are completed online. A total of 1693 expectant mothers/birthing parents and 731 partners from Dresden, Germany and nearby areas were included in the cohort. Detailed information on design, sample, and procedures can be found in Vollert et al. (2025) 48 . For the present study, data from T1 and T2 have been included.

Ethical approval for RESPECT was obtained from the Ethics Committee of the Technische Universität Dresden (No: SR-EK-331072022). The project was pre-registered at the Open Science Framework (OSF) registries: https://doi.org/10.17605/OSF.IO/CAQG7

### Sample

The present cohort consists of a community sample of (expectant) mothers/birthing parents and partners, primarily recruited at all maternity clinics and a freestanding birth center in Dresden, Germany. Recruitment took place from April 2023 to December 2024. Eligibility was determined via a screening questionnaire and required participants to

- be currently pregnant or have a pregnant partner,
- be at least 18 years old,
- reside in or plan childbirth in the Dresden area, and
- have sufficient German or English language skills.

Participation did not involve monetary compensation. Rather, participants were provided with small incentives upon study registration and prior the third and fourth assessment point.

**Fig. 1:**
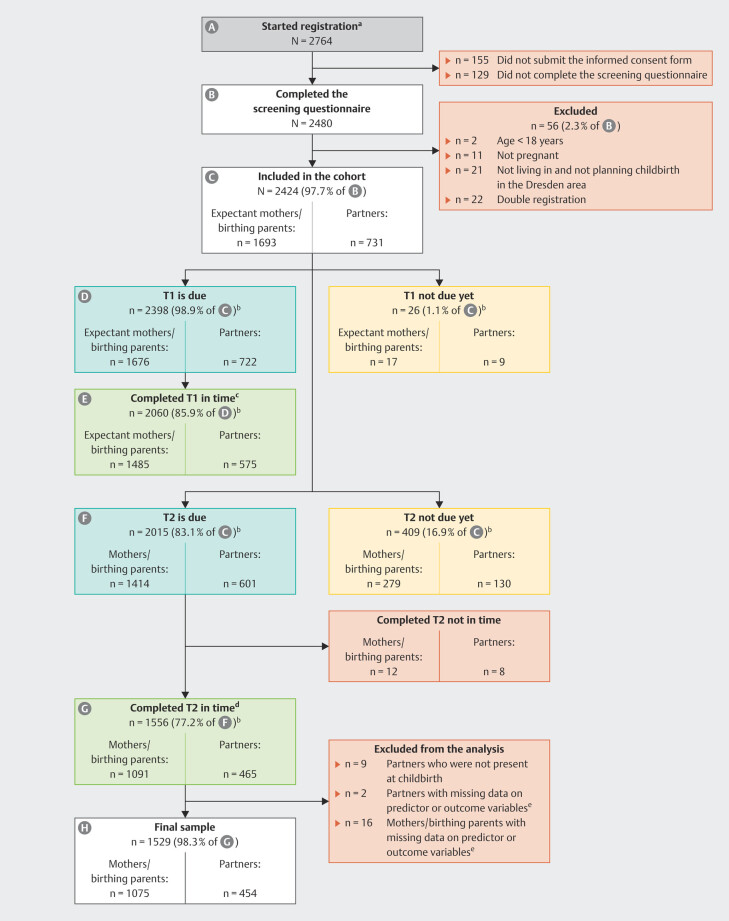
Flowchart of parents included in the current study. *Note.* T1 = during pregnancy (24 ^th^ week of gestation onwards); T2 = around eight weeks after anticipated birth date. Data for the current study was extracted on January 2, 2025 (version 5 of the quality-assured data files). ^a^ Means that they opened at least the study registration website on REDCap and proceeded to the informed consent form. ^b^ Prospective data collection ongoing. ^c^ Before the birth of the child. ^d^ Within 6 and 18 weeks after the actual birth date. ^e^ Includes mistreatment index, discrimination, subjective birth experience (W-DEQ, version B), CB-PTSD symptoms (City BiTS).

This study, based on version 5 of the quality-assured data files of the RESPECT study, includes data from mothers/birthing parents and partners who participate in RESPECT _PARENTS_ and completed at least the T2 assessment until January 2, 2025 (data export date). A total of n = 1414 mothers/birthing parents and n = 601 partners were initially contacted to schedule an appointment for the T2 interview. Of these, n = 1103 mothers/birthing parents and n = 473 partners participated in the interview. Fig. 1 illustrates the participant flow and retention rate. To ensure consistency, data from T1 were excluded if the online questionnaire had been completed after the child was born. Data from participants who did not complete T2 within the timeframe of six to 18 weeks postpartum were excluded, in order to gather comparable data regarding the time of reporting. Furthermore, data from mothers/birthing parents and partners who did not complete measures assessing the predictor or outcome variable and partners who were not present at childbirth were excluded from the analysis. The final sample for the analysis therefore consisted of n = 1075 mothers/birthing parents and n = 454 partners.

### Measures

**Instances of mistreatment** encountered during childbirth were assessed at T2 using the mistreatment questionnaire developed by Limmer et al. (2023) as part of a validated survey instrument for measuring disrespect and abuse during childbirth in Germany 6 19 . One original item addressing physical and sexual abuse was divided into two items, therefore addressing physical abuse and inappropriate sexual conduct separately, in accordance with the WHO typology of mistreatment during childbirth 1 . The adapted questionnaire consists of 13 items, addressing different categories of mistreatment (i.e., physical abuse, verbal abuse, sexual abuse, and failure to meet professional standards of care 1 ). Participants provided dichotomous responses in a yes-no format indicating whether or not they had experienced the events described by the items. If the item addressing interventions conducted without consent is answered positively, it lists possible interventions (e.g., episiotomy, injection, venous access), including an “other” category, to record the number and nature of specific interventions without consent in greater detail. The full instrument was employed for mothers/birthing parents. In this study, the instrument was adapted for partners by omitting five items as they did not physically experience childbirth and therefore could not have experienced some of the events themselves. The total number of affirmative responses was used to calculate a mistreatment index. A higher score on this index indicates a greater accumulation of mistreatment experiences during childbirth.

**Instances of discrimination** were recorded at T2 through an adapted version of the discrimination list that is also part of the instrument developed by Limmer et al. (2023) 19 . Each item began with the prompt, “When I had my baby, I felt that I was treated poorly by my midwife or doctor because of …” 19 . Following consultation with the RESPECT Advisory Board, the original discrimination list was adapted by adding seven items. In the original list, the following nine reasons for discrimination during childbirth were included: race/ethnicity/cultural background/language, sexual orientation/gender identity, handicap/chronic disease, HIV status, age, overweight, socioeconomic situation, type or lack of health insurance, differing opinion with caregivers about the care for oneself or the baby 19 . In the revised list, the following reasons were added: religious affiliation, mental illness, alcohol and/or drug use, acute COVID-19 infection, underweight, number of older children, being a single mother (only for mothers/birthing parents), other reasons. Other reasons were assessed using a subsequent open question format. Each item was encoded dichotomously in a yes-no format, in order to allow the reporting of multiple reasons. Perceived discrimination during childbirth was used as a dichotomous variable with values of 0 (in absence of affirmation of any reasons on the discrimination list) or 1 (if one or more reasons for discrimination were affirmed). The data concerning perceived discrimination during childbirth were dichotomized due to the fact that only a small number of participants exhibited characteristics that could potentially result in the identification of multiple reasons for discrimination. Consequently, the interpretation of a sum score for experiences of discrimination during childbirth would have been impracticable.

**Subjective birth experience** was assessed at T2 using the German version of the Wijma Delivery Expectancy/Experience Questionnaire (W-DEQ), version B 49 50 . The questionnaire assesses multiple dimensions of the childbirth experience, including cognitive aspects, emotional aspects, perceived control, and physical sensations. The scale entails 33 items in total that are answered on a 6-point Likert-type-scale, ranging from 0 to 5, presented with varying positive and negative response anchors (e.g., “extremely frightful” – “not at all frightful”). The total score of the instrument ranges from 0 to 165, indicating how positive or negative the childbirth was subjectively experienced. A higher score indicates a more negative experience, with a score of 85 or greater reflecting a very negative childbirth experience. All 33 items can be used for both parents, but the phrasing of the instructions for partners was adapted. The reliability for the W-DEQ, version B in the current study sample was good for both mothers/birthing parents (Cronbach’s α = 0.90) and partners (Cronbach’s α = 0.89).

**CB-PTSD symptoms** were assessed at T2 using the German version of the City Birth Trauma Scale (City BiTS) 51 52 . This instrument comprises 29 items aligning with the Fifth Edition of the Diagnostic and Statistical Manual of Mental Disorders (DSM-5) criteria for PTSD 53 . Two binary items are employed to assess the stressor criterion, asking whether the participant fears serious injury or death to either the mother or the child during or immediately after childbirth. Participants are asked to report their perceived symptoms over the previous week. Symptoms are assessed within two sections: the first section addresses childbirth‑related symptoms of PTSD (e.g., intrusion and avoidance), which are measured by ten items, while the second section addresses general PTSD symptoms (e.g., hyperarousal), which are also measured by ten items. Response options, indicating the frequency of occurrence of the symptoms, range from 0 (“not at all”) to 3 (“5 or more times”). Consequently, a total symptom score between 0 and 60 is derived, with higher scores expressing a higher severity of CB-PTSD symptoms. The additional items are used to record the onset, duration, distress, and impairment due to symptoms, if present, and whether the symptoms can be explained by other conditions (exclusion criteria) 52 . The reliability for the City BiTS in the current study sample was good for both mothers/birthing parents (Cronbach’s α = 0.84) and partners (Cronbach’s α = 0.74).

**Potential confounding variables** were identified based on prior research and included the following factors, associated with mistreatment and discrimination during childbirth, the subjective birth experience, and CB-PTSD symptoms: age 54 , socioeconomic status (SES) 55 , being a first-time parent 56 , fear of childbirth (FOC) 57 58 59 , pregnancy complications 27 57 , place of birth 60 61 (i.e., maternity clinic, freestanding birth center, or home birth), mode of birth 58 62 , birth complications 27 57 , pre-existing mental disorders 23 63 , including a diagnosis of depression, anxiety disorder or PTSD prior to pregnancy, and symptoms of depression, anxiety disorders, or CB-PTSD during pregnancy. Age, SES index, being a first-time parent, FOC, and symptoms of depression, anxiety and prior CB-PTSD were measured at T1 during pregnancy, while all other potential confounders were measured at T2 eight weeks after the anticipated birth date.

The socioeconomic status (SES) was calculated as an index based on educational qualification, equivalent net income situation, and occupational status according to Lampert et al., 2018 64 . The SES index was included in the analyses and can range from 3.0 to 21.0, with values up to 8.7 representing a low SES, values up to 16.9 representing a medium SES, and values from 17.0 onwards representing a high SES.

Fear of childbirth (FOC) was measured with the W-DEQ, version A 50 . It assesses FOC by focusing on the cognitive appraisal related to the forthcoming childbirth. Equivalent to version B of the W-DEQ, it consists of 33 items, each rated on a scale from 0 to 5, resulting in a total score that ranges from 0 to 165. Higher scores reflect a greater degree of FOC, with a cut-off score of 85 or higher indicating a significant level of FOC. The instrument was used for both expectant parents, with the same adjusted phrasing of the instructions for partners as in version B. In the current study sample, the reliability of the W-DEQ, version A was excellent (mothers/birthing parents: Cronbach’s α = 0.92, partners: Cronbach’s α = 0.88).

Pre-existing mental disorders, including diagnoses of depression, anxiety disorders, or PTSD prior to the current pregnancy, were assessed based on self-report and incorporated as dichotomous variables.

Symptoms of depression during pregnancy were assessed using the German version of the Edinburgh Postnatal Depression Scale (EPDS) 65 66 . It consists of ten items, each with four possible response options on a scale of 0 to 3, and measures symptoms experienced during the previous week. The total score ranges from 0 to 30, with a higher score indicating more severe symptoms of depression. A likely presence of a depressive disorder was indicated by means of a cut-off of 10 or more. The reliability in the study sample was good for both mothers/birthing parents (Cronbach’s α = 0.85) and partners (Cronbach’s α = 0.79).

Symptoms of anxiety during pregnancy were measured using the Generalized Anxiety Disorder 7 (GAD-7) 67 68 , a validated seven-item self-report questionnaire to screen for generalized anxiety disorder. The frequency of anxiety symptoms experienced over the past two weeks is rated on a scale from 0 (“not at all”) to 3 (“nearly every day”). The total score ranges from 0 to 21, with higher scores indicating greater severity of symptoms of anxiety. The reliability in the study sample was good for both mothers/birthing parents (Cronbach’s α = 0.81) and partners (Cronbach’s α = 0.84).

Symptoms of pre-existing CB-PTSD were assessed during pregnancy using the City BiTS 51 52 in expectant parents who already had at least one child at the time of study registration. Participants without a previous own childbirth experience or without any own children were assigned a score of 0, indicating the absence of CB-PTSD symptoms. The reliability of the instrument was good for both mothers/birthing parents (Cronbach’s α = 0.86) and partners (Cronbach’s α = 0.80) who completed the City BiTS.

Pregnancy complications were identified from the maternity record 69 and included 23 items (e.g., preeclampsia, gestational diabetes, cervical insufficiency). Mothers/birthing parents were asked whether they had experienced the complication. Experienced pregnancy complications were incorporated into the analyses using a sum score. The sum scores ranged from 0 to 53.97, with higher scores indicating a higher quantity and more severe pregnancy complications. The severity of each complication was rated on a scale from 1 to 4. These ratings were assigned by 15 experts in the field of obstetrics/midwifery, with the final score for each complication calculated as the average of all expert ratings. As partners were not asked to report on complications during pregnancy, this confounder was only included within the analyses for mothers/birthing parents.

Birth complications were assessed through self-report, capturing 19 commonly occurring complications (e.g., perineal tear, premature placental abruption, weakness of labor) in a yes-no format. The score for birth complications was developed following the same methodological approach as the score for pregnancy complications. The total scores range from 0 to 51.11, with higher scores indicating a higher quantity and more severe birth complications. Each item was rated on a scale from 1 to 4 by the experts. The mean value for each item was subsequently calculated, and the final score was derived by summing the values of the items that were affirmed by the mother/birthing parent. Complications during childbirth again were not assessed for partners and were therefore only included within the analyses for mothers/birthing parents.

### Data analysis

All analyses were performed using IBM SPSS Statistics (Version 29.0.2.0). First, data were examined for outliers. Univariate outliers were assessed using boxplots, applying a threshold of plus or minus three standard deviations. No data points had to be removed based on substantive plausibility considerations. In the multivariate outlier analysis using Mahalanobis distance, all discrimination cases were identified as outliers. However, given that the aim of this study was to analyze perceived discrimination during childbirth and its potential impact on the subjective birth experience and CB-PTSD symptoms, participants who experienced discrimination during childbirth were precisely the subject of interest and were therefore retained in the analysis sample. Attrition analyses were conducted to compare participant characteristics between completers and non-completers.

In cases where the number of missing values on the psychometric scales did not exceed 20%, missing values were imputed using the participants’ mean value of the respective scale.

Analyses were conducted separately for 1) mothers/birthing parents and 2) partners, once without confounding variables and once adjusted for the confounding variables. First, descriptive analyses were conducted and attrition analyses were performed using independent samples t-tests for continuous variables and Fisher’s exact tests for dichotomous variables to examine potential differences between completers and non-completers at T2. Second, bivariate Spearman correlations were calculated between all predictor variables, the mediator, outcome variable, and all potential confounders. Only those confounders which had a significant correlation with the outcome were included as confounders in the analyses. Third, a multiple linear regression was conducted to test the associations between the mistreatment index, discrimination during childbirth, and CB-PTSD symptoms, controlling for the statistically significant potential confounders.

Finally, a mediation analysis was conducted to investigate whether the subjective birth experience mediates the association between mistreatment during childbirth and CB-PTSD symptoms. The same analysis was repeated using discrimination during childbirth as the predictor. For all mediation analyses, the SPSS modeling tool PROCESS 4.3.1 70 was utilized, initially for unadjusted models and subsequently controlling for potential confounding variables in all models. The tool applies ordinary least squares regression to calculate unstandardized path coefficients for total, direct, and indirect effects in a mediation model. For the predictor mistreatment during childbirth, standardized regression coefficients are reported. For models with the predictor perceived discrimination, in which standardization was not feasible due to data scale limitations, unstandardized coefficients are presented instead. The mediation effect was identified using 95% confidence intervals for the indirect effects, with significance assumed if the intervals did not include zero. To obtain confidence intervals and inferential statistics, the tool employs bootstrapping with 5000 iterations, along with heteroscedasticity-consistent standard errors 71 . All analyses were performed at a significance level of p < 0.05 with corresponding 95% confidence intervals.

## Results

### Attrition analyses

Attrition analyses (see Supplementary Table **S1** , online) were conducted comparing sociodemographic and pregnancy-related characteristics of completers vs. non‑completers at T2, including all participants for which participation in the T2 interview was due by January 2, 2025. The analyses were conducted separately for mothers/birthing parents and partners. Among mothers/birthing parents, completers were more often first‑time parents (57.0% vs. 48.3%; Fisher’s exact test, p = 0.018), were older ( *t* (1410) = −3.39, p < 0.001, 95% CI [−1.75, −0.47]), reported higher mean SES index scores ( *t* (1314) = −7.21, p < 0.001, 95% CI [−2.05, −1.17]), and had lower levels of symptoms of depression ( *t* (1218) = 3.33, p < 0.001, 95% CI [0.49, 1.89]) compared to non-completers. Among partners, completers reported higher mean SES index scores ( *t* (536) = −4.44, p < 0.001, 95% CI [−2.24, −0.87]) and higher FOC ( *t* (428) = −2.25, p = 0.025, 95% CI [−10.45, −0.69]) compared to non-completers.

### Descriptive analyses

For the present study, the final sample comprised n = 1529 parents, including n = 1075 mothers/birthing parents (mean age: 32.12 years, SD = 4.93) and n = 454 partners (mean age: 34.23 years, SD = 5.40). Table 1 shows the sample sociodemographic and birth‑related characteristics for both mothers/birthing parents and partners. Results show that 56.9% of mothers/birthing parents and 68.5% of partners were expecting their first child. The SES index of participating expectant parents indicates an elevated SES compared to the general German population 64 . Consistent with the national average in Germany 72 , 96.8% of mothers/birthing parents gave birth in a maternity clinic, while 96.4% of partners attended childbirth in this setting. For the majority (92.7% of mothers/birthing parents and 97.8% of partners), the total W-DEQ, version B score was below the clinically relevant cut-off, indicating predominantly positive subjective birth experiences among both parents. Among mothers/birthing parents, n = 24 individuals (2.2%) met the criteria for CB-PTSD according to DSM-5, whereas among partners, n = 4 individuals (0.9%) met the criteria for CB-PTSD.

**Table:** **Table 1** Sample description.

	Mothers/birthing parents (n = 1075)		Partners (n = 454)	
**Sample characteristics at T1**	**M (SD)**	**Range**	**M (SD)**	**Range**
Parental age (in years)	32.12 (4.93)	18–45	34.23 (5.40)	20–54
Gestational age (in weeks)	33.01 (4.74)	24–42	33.20 (4.81)	25–42
SES index	16.22 (2.97)	6.5–21	16.71 (2.69)	6.4–21
Fear of childbirth (W-DEQ, version A score, 0–165)	65.11 (20.33)	10–134	58.66 (17.43)	6–101
Depressive symptoms (EPDS score, 0–30)	6.90 (4.72)	0–27	4.00 (3.55)	0–17
Anxiety symptoms (GAD-7 score, 0–21)	4.93 (3.62)	0–21	3.76 (3.37)	0–18
CB-PTSD symptoms (City BiTS score, 0–60)	8.61 (9.43)	0–60	3.86 (5.91)	0–37
	** n ^a^**	** % ^b^**	** n ^a^**	** % ^b^**
Relationship status				
In a permanent relationship	1003	98.0	395	99.7
Not in a permanent relationship	20	2.0	1	0.3
Mother tongue				
German ^c^	984	91.5	413	91.0
Other	91	8.5	41	9.0
First-time parent				
Yes	609	56.9	311	68.5
No	461	43.1	143	31.5
**Sample characteristics at T2**	**M (SD)**	**Range**	**M (SD)**	**Range**
Gestational week at birth	39.89 (1.76)	29–43	40.05 (1.70)	29–43
Subjective birth experience (W-DEQ, version B score, 0–165)	50.00 (22.58)	1–128	44.02 (18.68)	1–125
	** n ^a^**	** % ^b^**	** n ^a^**	** % ^b^**
Mode of birth				
Vaginal birth	746	69.4	314	69.3
Vaginal-operative birth ^d^	58	5.4	20	4.4
Planned cesarean section	113	10.5	51	11.3
Unplanned cesarean section	134	12.5	58	12.8
Emergency cesarean section	24	2.2	10	2.2
Place of birth				
Maternity clinic	1041	96.8	438	96.4
Birth center	15	1.4	8	1.8
Home birth	18	1.7	8	1.8
On the way	1	0.1	0	0
Pregnancy complications				
Yes	556	52.4	n.a.	n.a.
No	505	47.6	n.a.	n.a.
Birth complications				
Yes	945	88.1	n.a.	n.a.
No	128	11.9	n.a.	n.a.
Number of children born				
One	1044	97.1	443	97.6
Twins	31	2.9	11	2.4
*Note.* City BiTS = City Birth Trauma Scale; EPDS = Edinburgh Postnatal Depression Scale; GAD7 = Generalized Anxiety Disorder Scale-7; SES = Socioeconomic status; W-DEQ version A/B = Wijma Delivery Expectancy/Experience Questionnaire. T0 = at study registration/screening; T1 = during pregnancy; T2 = 8 weeks after the expected birth date. ^a^ n varies slightly due to missing data of some participants. ^b^ Valid percent. ^c^ Including German only and German plus another language. ^d^ With forceps or vacuum extraction.				

### Mistreatment and discrimination during childbirth

Table 2 provides a detailed overview of instances of mistreatment during childbirth reported by mothers/birthing parents and partners. At least one instance of mistreatment during childbirth was reported by 25.8% of mothers/birthing parents, with the number of reported experiences ranging from 0 to 9. The most frequently mentioned instance of mistreatment was the exertion of fundal pressure with hands or forearms to assist in birth (9.1%), followed by physical abuse (4.9%) and the refusal of assistance when urgently needed (4.7%). Among partners, at least one instance of mistreatment during childbirth was reported by 6.8% of participants, with the number of experiences ranging from 0 to 4. The most frequently reported instances of mistreatment were lack of timely support (2.9%) and refusal of assistance when needed (2.4%).

**Table:** **Table 2** Perceived mistreatment during childbirth in mothers/birthing parents and partners.

	Mothers/birthing parents (n = 1075)		Partners (n = 454)	
	**n**	**%**	**n**	**%**
No instances of mistreatment reported	798	74.2	423	93.2
At least one instance of mistreatment reported	277	25.8	31	6.8
Your private or personal information was shared without your consent.	7	0.7	0	0
Your physical privacy was violated (i.e., being uncovered or having people in the delivery room without your consent).	32	3.0	–	–
A health care provider (doctor, midwife, or nurse) shouted at or scolded you.	41	3.8	6	1.3
A midwife, a doctor, or a nurse threatened to withhold treatment or to force you to accept treatment that you did not want.	37	3.4	–	–
A midwife, a doctor, or a nurse threatened you in any other way (e.g. with danger of live for your baby).	14	1.3	3	0.7
A midwife, a doctor, or a nurse made disparaging remarks against you.	33	3.1	7	1.5
Health care provider(s) ignored you, refused your request for help, or failed to respond to requests for help in a reasonable amount of time.	50	4.7	11	2.4
The health care providers had no time for you when you needed help.	42	3.9	13	2.9
You experienced physical abuse (including painful vaginal examination, insufficient anesthesia for an episiotomy, aggressive physical contact, etc.	53	4.9	–	–
You experienced inappropriate sexual conduct.	1	0.1	–	–
A midwife, a doctor, or a nurse conducted fundal pressure with the hands or forearms to help the baby out.	98	9.1	–	–
Interventions (e. g., episiotomy, cesarean section, oxytocin infusion, amniotomy, drug injection, venous access) were conducted without your consent.	34	3.2	–	–
Which intervention(s) was/were involved?				
Episiotomy	18	1.7		
Amniotomy	3	0.3		
Vaginal examination	3	0.3		
Venous access	3	0.3		
Oxytocin infusion	2	0.2		
Drug injection	2	0.2		
Kristeller maneuver or similar (applying pressure on the top of the abdomen with hands)	2	0.2		
Continuous/permanent CTG (cardiotocography)	2	0.2		
Cervical dilator to induce labor	1	0.1		
Balloon catheter to induce labor	1	0.1		
Insertion of a bladder catheter	1	0.1		
Other relevant and thematically similar experiences during childbirth ^a^	18	1.7	3	0.7
	**M (SD)**	**Range**	**M (SD)**	**Range**
Number of mistreatment experiences	0.43 (0.94)	0–9	0.09 (0.40)	0–4
*Note* . Possible interventions included in the questionnaire, but not reported by the mothers/birthing parents: Membrane sweep, also called cervical stripping; Puncture of the fetal scalp for blood sampling (fetal blood analysis); Cesarean section; Forceps delivery; Vacuum-assisted vaginal delivery; Umbilical cord traction; Enema. ^a^ Similar experiences included, e.g., no free choice of birth position or inadequate anesthesia.				

The frequency of perceived reasons for discrimination during childbirth is presented in Table 3 . Among mothers/birthing parents, n = 42 (3.9%) reported instances of discrimination for at least one reason, as did n = 16 (3.5%) of partners. The most prevalent reason for discrimination during childbirth were differences in opinions regarding the optimal care for the (expectant) mother or the baby between caregivers and (expectant) parents (2.5% of mothers/birthing parents, 2.9% of partners). The second most prevalent reason among mothers/birthing parents was mental illness and among partners gender identity. Other reasons indicated by the participants that were not covered by the pre-specified discrimination list included, for example, having scars on the arms or being a nurse.

**Table:** **Table 3** Perceived discrimination during childbirth in mothers/birthing parents and partners.

	Mothers/birthing parents (n = 1075)		Partners (n = 454)	
	**n**	**%**	**n**	**%**
No	1033	96.1	438	96.5
Yes	42	3.9	16	3.5
... because of my religious affiliation.	1	0.1	1	0.2
… because of my sexual orientation and/or gender identity.	0	0	3	0.7
... because of my handicap/chronic disease.	5	0.5	0	0
... because of my mental illness.	8	0.7	0	0
... because of my age.	6	0.6	1	0.2
... because of my overweight.	5	0.5	0	0
... because of the number of children I already have.	3	0.3	0	0
... because I am or will be a single mother.	1	0.1	0	0
... because of my type of health insurance or lack of insurance.	2	0.2	0	0
... because of a difference in opinion with my caregivers about the right care for myself or my baby (my babies).	27	2.5	13	2.9
Other reason	4	0.4	1	0.2
	**M (SD)**	**Range**	**M (SD)**	**Range**
Number of reasons for discrimination experiences	0.06 (0.34)	0–5	0.04 (0.24)	0–3
*Note.* Possible reasons included in the questionnaire, but not reported by participants: race, ethnicity, cultural background or language; alcohol and/or drug use; acute COVID-19 infection; HIV status; underweight; socioeconomic situation.				

### Associations between mistreatment and discrimination during childbirth, the subjective birth experience, and CB-PTSD symptoms

Spearman correlation analyses were conducted (for mothers/birthing parents see Supplementary Table **S2** , online, for partners see Supplementary Table **S3** , online). Within the mothers’/birthing parents’ sample, significant correlates of more severe CB-PTSD symptoms were being a first-time parent, lower SES, higher FOC, higher depressive and anxiety symptoms during pregnancy, more pregnancy and birth complications, a cesarean section as birth mode, and diagnoses of depression, anxiety, or PTSD prior to the pregnancy. Within the partners’ sample, significant correlates of more severe CB-PTSD symptoms were younger age, being a first-time parent, lower SES, higher FOC, higher depressive and anxiety symptoms during pregnancy, a cesarean section as birth mode, and a diagnosis of depression prior to the pregnancy.

Second, multiple linear regression models were used to examine the associations between mistreatment and discrimination during childbirth with CB-PTSD symptoms. For mothers/birthing parents, in the unadjusted model without confounders, more mistreatment experiences (β = 0.30, p < 0.001) and experienced discrimination during childbirth (β = 0.10, p = 0.003) predicted higher levels of CB-PTSD symptoms. In the model including confounders, more mistreatment experiences during childbirth predicted significantly more severe CB-PTSD symptoms (β = 0.21, p < 0.001). The same applied to the effect of discrimination during childbirth (β = 0.09, p = 0.008). For partners, more mistreatment experiences during childbirth (β = 0.11, p = 0.043) and perceived discrimination during childbirth (β = 0.18, p < 0.001) were significantly associated with more severe CB-PTSD symptoms in the unadjusted model without confounders. In the model including confounders, both mistreatment (β = 0.06, p = 0.273) and discrimination (β = 0.11, p = 0.068) during childbirth were no longer significant predictors for more severe CB-PTSD symptoms.

#### Mediation analysis among mothers/birthing parents

**Fig. 2:**
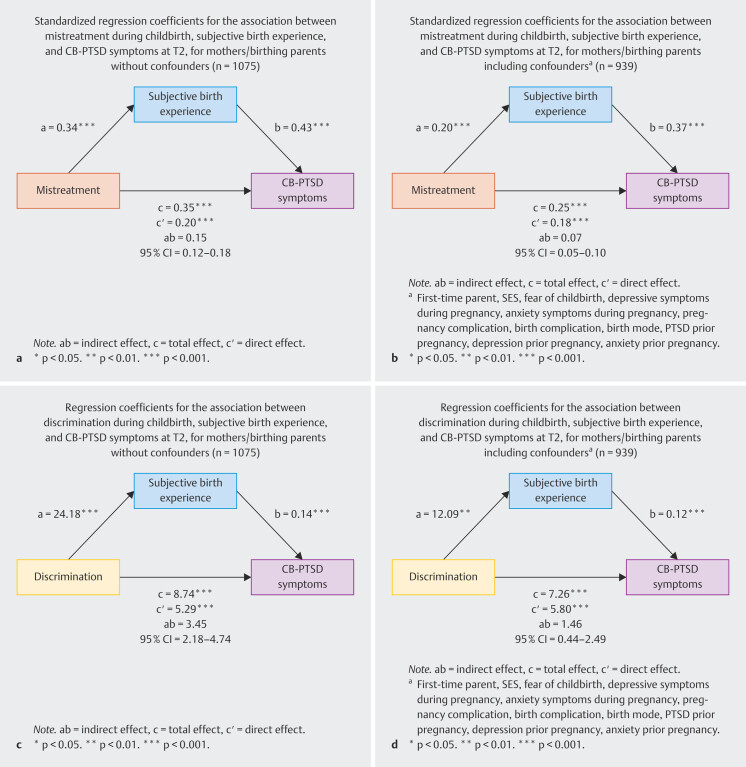
Standardized regression coefficients for the association between discrimination during childbirth, mistreatment during childbirth, subjective birth experience, and CB-PTSD symptoms at T2 for mothers/birthing parents.

In the mediation model (see Fig. 2 **a** ) for mothers/birthing parents, more mistreatment experiences significantly predicted a more negative subjective birth experience (β *_a_* = 0.34, p < 0.001), and a more negative subjective birth experience in turn significantly predicted more severe CB-PTSD symptoms (β *_b_* = 0.43, p < 0.001). The total effect of mistreatment experiences during childbirth on CB‑PTSD symptoms was significant (β *_c_* = 0.35, p < 0.001), and the direct effect remained significant, with a significant completely standardized indirect effect *ab* = 0.15, 95% CI [0.12, 0.18], indicating that the subjective birth experience acted as a partial mediator.

When including confounding variables into the model (see Fig. 2 **b** ), more mistreatment experiences continued to significantly predict a more negative subjective birth experience (β *_a_* = 0.20, p < 0.001), which in turn predicted significantly more severe CB-PTSD symptoms (β *_b_* = 0.37, p < 0.001). The total effect of mistreatment experiences during childbirth on CB-PTSD symptoms remained significant (β *_c_* = 0.25, p < 0.001). The significant direct effect and a significant completely standardized indirect effect *ab* = 0.07, 95% CI [0.05, 0.10] indicated partial mediation through the subjective birth experience.

As shown in Fig. 2 **c** , perceived discrimination during childbirth significantly predicted a more negative subjective birth experience ( *a* = 24.18, p < 0.001), which in turn significantly predicted more severe CB-PTSD symptoms ( *b* = 0.14, p < 0.001). The total effect was significant ( *c* = 8.74, p < 0.001), as were the direct effect and the indirect effect *ab* = 3.45, 95% CI [2.18, 4.74], indicating partial mediation.

When controlling for confounding variables (see Fig. 2 **d** ), the association between perceived discrimination and the subjective birth experience remained significant ( *a* = 12.09, p = 0.005). In turn, a more negative subjective birth experience still significantly predicted more severe CB-PTSD symptoms ( *b* = 0.12, p < 0.001). The total effect was significant ( *c* = 7.26, p < 0.001) as were both the direct effect and the indirect effect *ab* = 1.46, 95% CI [0.44, 2.49], indicating partial mediation.

#### Mediation analysis among partners

**Fig. 3:**
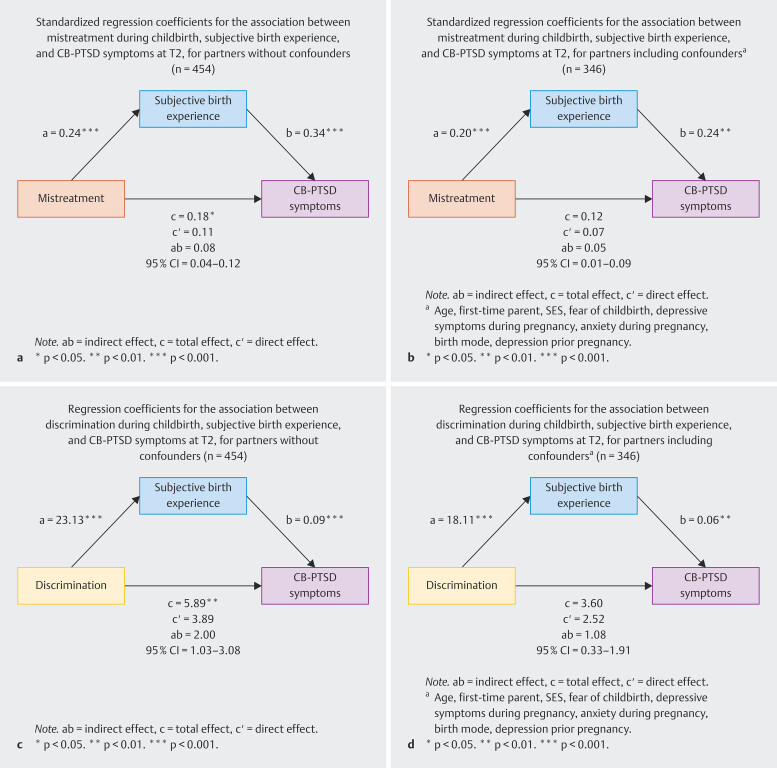
Standardized regression coefficients for the association between discrimination during childbirth, mistreatment during childbirth, subjective birth experience, and CB-PTSD symptoms at T2 for partners.

In the model including the mistreatment index score, the subjective birth experience, and CB-PTSD symptoms in the partner sample (see Fig. 3 **a** ), more mistreatment experiences significantly predicted a more negative subjective birth experience (β *_a_* = 0.24, p < 0.001), which in turn significantly predicted more severe CB-PTSD symptoms (β *_b_* = 0.34, p < 0.001). The total effect (β *_c_* = 0.18, p = 0.046) was significant. In the mediation analysis, the effect was partially mediated by the subjective birth experience with a significant completely standardized indirect effect *ab* = 0.08, 95% CI [0.04, 0.12].

When adding the confounding variables to the model (see Fig. 3 **b** ), more mistreatment experiences during childbirth continued to significantly predict a more negative subjective birth experience (β *_a_* = 0.20, p < 0.001), which in turn significantly predicted more severe CB-PTSD symptoms (β *_b_* = 0.24, p = 0.002). There was no significant total effect of mistreatment experiences during childbirth on CB-PTSD symptoms (β *_c_* = 0.12, p = 0.155), but the completely standardized indirect effect remained significant *ab* = 0.05, 95% CI [0.01, 0.09].

The experience of discrimination during childbirth was found to significantly predict a more negative subjective birth experience ( *a* = 23.13, p < 0.001; see Fig. 3 **c** ), which in turn significantly predicted more severe CB-PTSD symptoms ( *b* = 0.09, p < 0.001). Perceived discrimination during childbirth significantly predicted more severe CB-PTSD symptoms in total ( *c* = 5.89, p = 0.009), the indirect effect of the subjective birth experience as mediator was significant *ab* = 2.00, 95% CI [1.03, 3.08].

When including confounding variables to this model (see Fig. 3 **d** ), the association between discrimination during childbirth and subjective birth experience was still significant ( *a* = 18.11, p < 0.001), and a more negative subjective birth experience was still found to significantly predict more severe CB-PTSD symptoms ( *b* = 0.06, p = 0.002). Discrimination during childbirth was not a significant predictor for CB-PTSD symptoms anymore ( *c* = 3.60, p = 0.131), but the effect was mediated by subjective birth experience *ab* = 1.08, 95% CI [0.33, 1.91].

## Discussion

The aim of this study was to investigate the prevalence of mistreatment and discrimination during childbirth for both parents and their associations with the subjective birth experience and CB-PTSD symptoms. The findings of this study suggest that experiences of mistreatment and discrimination during childbirth are prevalent among both parents and experiences of mistreatment and discrimination during childbirth are associated with more CB-PTSD symptoms, with this association being partially mediated by the subjective birth experience in both parents. Among partners, in the models adjusted for confounding variables, the path between mistreatment and discrimination during childbirth and CB-PTSD symptoms became non-significant. All other paths remained unaffected, thereby preserving the mediating effect of the subjective birth experience. In light of the limited research findings for partners compared to mothers/birthing parents in the field of perinatal health in general, the results reported for partners are particularly interesting. Given that partners are often confined to an observational (passive) role during childbirth rather than being seen as active participants, this study shows that the quality of interactions with obstetric health care staff during childbirth do also affect their experiences and mental health.

### Prevalence of mistreatment and discrimination during childbirth

The reported rates of mistreatment and discrimination underscore the pervasiveness of these experiences.

The prevalence of mistreatment during childbirth among mothers/birthing parents of 25.8% represents a relevant issue in Germany. However, a Dutch study and another German study reported even higher prevalence rates of mistreatment during childbirth among mothers/birthing parents. In the non-representative German study experiencing mistreatment during childbirth was reported by 77.6% of the women 19 , in a Dutch study, 54.4% of women reported experiencing mistreatment during childbirth 21 . Conversely, a comparatively low percentage of mothers in a French study, specifically 10.56%, reported experiencing mistreatment during childbirth 22 . These substantial discrepancies complicate drawing definitive conclusions about the quality of obstetric health care in the respective countries, regarding obstetric mistreatment. Instead, the studies varied in the composition of the samples, including differences in recruitment strategies and sample sizes. Furthermore, the assessment tools used differed in the number of experiences captured. A larger number of questions in the present study might have captured a broader range of mistreatment experiences during childbirth. With 9.1%, fundal pressure was the most commonly reported reason for mistreatment during childbirth. This intervention is not recommended in the guideline *Vaginal birth at term* but is included as a possibility 73 , although it is a controversial intervention internationally 74 75 76 77 78 . The item was originally included in the assessment tool developed by Limmer et al. (2023). Furthermore, it is frequently perceived as intrusive by mothers/birthing parents 77 78 79 . Therefore, it remained in the analyses of this study.

Despite the fact that the reported prevalence of mistreatment during childbirth is lower among partners, with 6.8% compared to 25.8% among mothers/birthing parents, it represents an unexpected concern for partners due to their lack of physical involvement in the birth process. This underscores that mistreatment during childbirth is not only a concern for mothers/birthing parents but also profoundly affects partners. Furthermore, experiences of mistreatment during childbirth are relevant for partners particularly in terms of the negative impact on potential outcomes, such as the subjective birth experience. To the best of our knowledge, there are no comparable quantitative studies focusing on partners.

Discrimination during childbirth was reported by 3.9% of mothers/birthing parents and 3.5% of partners. The most prevalent perceived reason for discrimination reported by both parents was “because of a difference in opinion with my caregivers about the right care for myself or my baby” 19 . The prevalence of discrimination experiences found in this study is significantly lower compared to another German study by Limmer et al. (2023) which found a prevalence of 49.6%. Both studies utilized the same assessment tool except for the modifications described above; however, the sample in the non-representative study by Limmer et al. (2023) is not comparable to the community-based sample in this study. Our study sample was drawn from Dresden and the surrounding areas and was recruited before childbirth, while the sample in Limmer et al. (2023) was drawn from across Germany and recruited online after childbirth, specifically aiming at validating a tool to assess mistreatment in Germany. Additionally, the assessment in this study was conducted eight weeks after childbirth, while Limmer et al. (2023) assessed discrimination and mistreatment experiences during childbirth up to several years after childbirth, which might have influenced the participants’ recall 80 . However, having a different opinion about the right care was the most commonly reported reason for discrimination in both studies, suggesting that problems with shared decision-making between parents and obstetric health care staff may be of particular concern regarding obstetric health care practices in Germany. These findings are consistent with the results of a study conducted in the United States where women reported feeling discriminated against, particularly when their perspectives on their care or the care of their child differed from those of the obstetric health care staff 13 . Similarly, the *Giving Mothers a Voice study* 6 demonstrates that discrimination, as a form of mistreatment during childbirth, constitutes a significant issue within the field of obstetric health care, contributing to a poorer quality of medical health care.

To the best of our knowledge, no previous research has systematically examined partners’ experiences of mistreatment during childbirth, precluding direct comparisons with existing literature. Nevertheless, qualitative research suggests that discrimination may be a concern for partners within the context of childbirth and constitutes an issue in obstetric health care 81 . Our results extend these qualitative findings by demonstrating that, supported by our systematically collected prevalence data, partners are not (passive) bystanders during childbirth but subjects who can also become exposed to mistreatment and discrimination during childbirth. By considering this in our study and drawing attention to both parents’ perspective, this study offers insights into an underexplored aspect of obstetric health care.

### Association between mistreatment and discrimination during childbirth and the subjective birth experience

Mistreatment during childbirth was a significant predictor of a more negative subjective birth experience at eight weeks postpartum in both parents, which is in line with previous research 21 27 80 82 83 . This effect remained significant even after adjusting for risk factors for a negative subjective birth experience, such as mode of birth, birth complications and interventions, and FOC 27 . Therefore, the present study demonstrates experiences of mistreatment and discrimination during childbirth are significant predictors of a negative subjective birth experience over and above these risk factors for both parents. Such behaviors by health care staff may contribute to adverse outcomes, thereby compounding objective risk factors 84 . Respectful obstetric health care that actively counteracts the occurrence of mistreatment during childbirth can contribute to and is crucial for a more positive subjective birth experience 2 85 86 . While respectful obstetric care cannot be interpreted as the direct opposite of mistreatment during childbirth, it has been shown to contribute to a reduction of adverse experiences 83 87 88 .

Therefore, in addition to objective birth-related factors (e.g., medical interventions, complications), expectant parents’ perceptions of obstetric health care during childbirth play an important role in shaping the subjective birth experience. Care-related aspects may even exert a more substantial effect than other factors, as the behavior of obstetric health care staff can mitigate the adverse effects of a challenging or highly medicalized birth 24 89 . This finding is not limited to mothers/birthing parents but also applies to partners 26 90 91 92 .

### Association between the subjective birth experience and CB-PTSD symptoms

In line with previous research 29 45 93 , a more negative subjective birth experience was a significant predictor of more severe CB-PTSD symptoms eight weeks postpartum in all models. A negative or traumatic subjective birth experience can result in the development of CB-PTSD symptoms as it may involve feelings of fear, helplessness, or loss of control, all of which are critical components of traumatic experiences 51 53 . These emotions, combined with perceived or actual threats to life or health during childbirth, whether to the mother/birthing parent or the child(ren) can overwhelm parents’ coping abilities, leaving them vulnerable to the development of CB-PTSD symptoms 51 .

Nevertheless, the observed associations appeared stronger among mothers/birthing parents. This finding indicates that the impact of the subjective birth experience on CB-PTSD symptoms in mothers/birthing parents may be amplified by their physical and emotional involvement during childbirth, in addition to the emotional aspect, which is also shared by partners. Furthermore, the clinical focus of obstetric health care staff is primarily directed toward mothers/birthing parents, which may result in partners receiving less attention, both in terms of positive attention and negative experiences such as mistreatment and discrimination during childbirth 94 95 . Social expectations placed on mothers/birthing parents regarding caring for their child, may impair their ability to cope with a traumatic birth experience and may further contribute to the development of CB-PTSD 96 97 . Among mothers/birthing parents, our findings align with previous research, where a more negative subjective birth experience predicted more severe CB-PTSD symptoms 45 98 99 . Consistent with these findings, similar associations among fathers were reported 98 99 .

Another possible explanation for the difference between mothers’/birthing parents’ and partners’ results could be that in our study, partners generally reported a more positive subjective birth experience on average, resulting in a reduced number of cases with extremely negative subjective birth experiences. Conversely, the reduced variance in the severity of the subjective birth experiences among partners could potentially contribute to weaker associations with more severe CB-PTSD symptoms within this group. However, extant research has demonstrated that fathers appear to be less affected by CB-PTSD than mothers 96 . McNab et al. (2022) found that effective communication between fathers and midwives enhanced fathers’ subjective birth experience, thereby reducing the risk of developing CB-PTSD 91 . Additionally, they demonstrated that fathers who experienced feelings of isolation during birth were more likely to report negative subjective birth experiences, which, in turn, increased their likelihood of developing CB-PTSD.

### Association between mistreatment and discrimination during childbirth, and CB-PTSD symptoms, and the mediating role of the subjective birth experience

Among mothers/birthing parents, experiencing mistreatment or discrimination during childbirth was a significant predictor of more severe CB-PTSD symptoms, with the subjective birth experience mediating this effect. These findings are comparable with prior research conducted in low-income countries 100 , as well as in middle- and high‑income countries 22 42 101 , showing that experiences of mistreatment during childbirth are indeed associated with negative effects on mental health, including more severe CB-PTSD symptoms. To the best of our knowledge, our study is the first to investigate the potential mediating effect of the subjective birth experience on the association between mistreatment and discrimination during childbirth and CB-PTSD symptoms in parents. However, the study by Dekel et al. (2017) shows that both a negative subjective birth experience and a lack of support during childbirth are predictors of more severe CB-PTSD symptoms in mothers/birthing parents 42 . In this study, inadequate support from obstetric health care staff is addressed by two items of the mistreatment index 19 , suggesting that experiences of mistreatment and a negative subjective birth experience both play a role in this association. A meta-analysis by Ayers et al. (2016) further supports the finding that, in addition to the subjective birth experience, the quality of support provided by obstetric health care staff during childbirth, particularly poor or insufficient support, is a significant predictor of the development of CB-PTSD symptoms 45 . Additionally, a more detailed examination of this association is provided in a Chinese study, which indicates that support from a doula, a trained professional providing additional emotional support during childbirth, not only positively influences the subjective birth experience of women, but also directly reduces the occurrence of CB-PTSD symptoms, with the subjective birth experience functioning as a mediator in this relationship 102 . Taken together, the current study and the aforementioned studies highlight the potential detrimental effects of lack of support during childbirth on both, the subjective birth experience and the development of future mental health disorders in mothers/birthing parents.

Among partners, there were no significant direct associations between mistreatment or discrimination during childbirth and CB-PTSD symptoms, but a mediating effect of the subjective birth experience was found. To the best of our knowledge, there are no quantitative studies investigating experiences of mistreatment and discrimination during childbirth among partners, and as a result, no other analyses are available regarding their associations with the subjective birth experience or CB-PTSD symptoms. Nevertheless, a qualitative study by Edwards et al. (2020) documents instances of discrimination during childbirth among partners, but it does not analyze the subsequent effects on their subjective birth experience and the development of CB-PTSD symptoms 81 . Due to the lack of the physical aspect of childbirth and the lower possibility to experience physical abuse, experiences of mistreatment and discrimination during childbirth may have a less direct impact on the development of CB-PTSD symptoms in partners. Additionally, the fact that partners generally reported fewer experiences of mistreatment during childbirth may have diminished the strength of the association with CB‑PTSD symptoms. Nonetheless, partners may experience psychological distress by witnessing a traumatic childbirth or mistreatment during childbirth by obstetric health care staff, which may affect their mental health. Therefore, it is important to acknowledge the psychological impact of such experiences and to offer adequate support 103 104 .

The available literature suggests that the quality of the subjective birth experience is associated with a variety of factors, including the quality of the medical care during childbirth 27 57 59 105 . In addition, the emotional environment during childbirth, characterized by obstetric health care staff behaviors ranging from support or mistreatment, can also have an impact on the subjective birth experience 106 107 . It is therefore essential to improve the care provided by obstetric health care staff during childbirth, with a particular focus on the psychological well-being of both parents.

### Strengths and limitations

This study has several strengths. An advantage is the implementation of the T2 assessment as a structured telephone interview, which proved to be a more practical method for parents in the postpartum period compared to online questionnaires. This approach aligns with previous studies that conducted assessments shortly after birth 108 109 . By studying a large sample of German mothers/birthing parents and partners, this study contributes to the limited evidence on mistreatment and discrimination during childbirth in Germany, an area that is underexplored, as most research focuses on low‑income countries 110 . To our knowledge, this is the first study to assess the prevalence of mistreatment and discrimination during childbirth within a community sample of mothers/birthing parents in Germany, and the first study to include partners addressing a gap in the literature regarding mistreatment and discrimination during childbirth from their perspective. Previous studies have mostly focused on pregnancy and birth complications or pre‑existing mental health disorders as risk factors for CB-PTSD symptoms 34 111 , often neglecting the role of interactions with obstetric health care staff. As such interactions can be actively modified by providers, the results of this study are particularly useful to develop care approaches that fit the needs of families. Additionally, this study is the first to examine the relationship between mistreatment and discrimination during childbirth, CB-PTSD, as well as the mediating role of the subjective birth experience for both mothers/birthing parents and partners. To assess the key constructs of this study, including mistreatment and discrimination during childbirth, the subjective birth experience, and CB-PTSD symptoms, only validated instruments were used, and all analyses were controlled for potential confounders.

However, it is imperative to also acknowledge the limitations of this study. While some of the confounders, including sociodemographic characteristics, prenatal mental health, and FOC were assessed during pregnancy, the key variables of this study (mistreatment and discrimination during childbirth, subjective birth experience, and CB-PTSD symptoms) were measured concurrently at the assessment point eight weeks postpartum. This approach has certain limitations, although the temporal sequence of events is clear. Mistreatment and discrimination during childbirth take place precisely during birth, while the subjective birth experience, shaped by all aspects of the birth, forms afterwards. CB‑PTSD symptoms manifest after birth according to the DSM-5 criteria. However, it is important to note that CB-PTSD symptoms may have influenced parents’ recall of their subjective birth experience, potentially introducing a bias 112 . Additionally, assessing the subjective birth experience eight weeks after childbirth may also entail the risk of recall bias, as memories of the childbirth may diverge from the actual events. However, some studies suggest that women tend to rate their overall birth experience more positively in the immediate postpartum period than at follow-up weeks or months later, because of initial feelings of relief at having overcome labor and pain and having a healthy baby 80 113 . This initial evaluation of the childbirth can become less positive when mothers had some time to reflect the birth process, to become aware of unmet expectations, and to integrate more negative aspects, including negative care‑related aspects. Against this background, we decided for an assessment eight weeks after childbirth when routines have become established and new parents are less burdened by a telephone interview compared to a point in time shortly after birth. With regard to the assessment of mistreatment and the subjective birth experience, there may be some possible overlap, as both the W-DEQ, version B and the mistreatment questionnaire capture aspects of childbirth experiences. However, the mistreatment questionnaire only documents specific behaviors of obstetric health care staff that constitute mistreatment, whereas the W-DEQ, version B assesses the subjective emotional appraisal of birth, including fear, sense of control, and feelings of safety. Thus, mistreatment during childbirth is considered as a determinant of the subjective birth experience (or a risk factor for a more negative birth experience) which has been the focus in the present study.

Furthermore, the sample of this study was not fully representative for the German population. Firstly, the study took place only in a limited area around the city of Dresden, which differs especially in cesarean section rates from other areas in Germany 114 . Secondly, attrition analysis revealed that non‑completers had a lower SES and, among mothers/birthing parents, higher levels of symptoms of depression during pregnancy. Additionally, the study sample exhibited a higher level of education and a higher SES compared to the general population in Germany 115 , which could contribute to an underrepresentation in the prevalence and severity of mistreatment and discrimination experiences during childbirth, as well as of CB-PTSD symptoms. The limited socioeconomic diversity may restrict the generalizability of our findings, as previous research suggests that women and families with lower SES (or markers of low SES such as low education, low income, or no health insurance) are more frequently exposed to mistreatment (or certain forms of mistreatment) and discrimination during childbirth 6 23 82 116 , and may also be at elevated risk for adverse psychological outcomes including CB-PTSD symptoms 117 . In contrast, the relatively high SES in our sample may have contributed to more positive subjective birth experiences and a lower incidence of CB-PTSD symptoms. Elevated educational attainment is frequently associated with enhanced health literacy, potentially affecting the awareness, reporting, and coping strategies related to traumatic events during childbirth 118 119 . Furthermore, it is possible that individuals with more severe CB-PTSD symptoms were less inclined to participate in the T2 interview due to the avoidance behaviors commonly associated with PTSD, such as evading reminders of the traumatic event, potentially leading to an underestimation of CB-PTSD symptoms among parents.

The recruitment strategy, focusing on Dresden and its surrounding areas, a region with fewer immigrants compared to other large cities in Germany 120 , and the requirement for participants to speak English or German likely restricted the diversity of the sample, reducing its ability to fully reflect varied experiences of mistreatment and discrimination during childbirth. Additionally, mothers/birthing parents in the study were relatively healthy, with only few participants experiencing severe pregnancy or birth complications. The cohort also exhibited relatively low levels of depression, anxiety, and CB-PTSD symptoms during pregnancy, with mean scores falling below clinically relevant cut-offs. Consequently, the findings of this study may not be generalizable to populations with higher levels of psychological distress or more complex obstetric histories. Nonetheless, the study provides valuable general insights, particularly for a community sample, into the role of mistreatment and discrimination during childbirth and their associations with the subjective birth experience and CB-PTSD symptoms.

### Implications for future research and clinical practice

Although initial studies provide some insights, there is a significant gap in quantitative evidence on the prevalence of mistreatment and discrimination during childbirth, particularly in high‑income countries. While qualitative research on this topic has been increasing, quantitative studies, especially those addressing the experiences of partners, remain limited 35 121 . However, the rates of mistreatment and discrimination found in this study underscore the pervasiveness of these experiences. Therefore, future research should prioritize investigating both the prevalence and psychological consequences of mistreatment and discrimination during childbirth for mothers/birthing parents and partners. Furthermore, reliable and consistent reporting of cases of mistreatment during childbirth is imperative to raise awareness and reduce the number of such adverse experiences 6 .

The present study focused on the immediate postpartum period, whereas in future studies it is important to examine whether the impact on psychological outcomes, including CB-PTSD symptoms, persists over time. The implementation of longitudinal study designs is crucial, as they will offer valuable insights into the enduring implications of adverse childbirth experiences on parental mental health and potentially also parent–infant interactions 122 .

Beyond psychological outcomes, subsequent research should investigate other potential outcomes impacted by mistreatment and discrimination during childbirth, such as physical health complications, breastfeeding experiences, or postpartum health care utilization 82 123 124 .

The study results indicate that experiencing mistreatment and discrimination during childbirth is associated with a more negative subjective birth experience also among partners. However, mistreatment and discrimination during childbirth did not directly predict more CB-PTSD symptoms in this group. Further research is required to elucidate the specific mechanisms underlying this relationship and to examine how psychological responses to childbirth experiences differ between mothers/birthing parents and partners 125 . In addition, future studies may benefit from the incorporation of a more diverse and heterogeneous sample with regard to sociodemographic and socioeconomic characteristics as well as clinical impairment of mental health in order to capture a broader range of childbirth experiences, better reflect structural inequalities in perinatal health care, and enhance generalizability of the results.

To minimize the occurrence of mistreatment and discrimination during childbirth, obstetric health care facilities can implement programs such as bias-awareness training 126 , patient‑centered care 127 , and culturally responsive care models 128 129 . While person‑centered care is not a complete prevention strategy for mistreatment and discrimination during childbirth, it has been identified as an effective model for reducing such events 130 131 132 . These approaches can promote sensitive and inclusive practices among obstetric health care staff. This responsibility extends beyond obstetrics and the medical sector, representing a challenge for society as a whole.

Given that CB-PTSD can occur in both parents following childbirth 35 , it is crucial to acknowledge the condition and ensure its appropriate identification and support. Implementing routine postpartum screening for CB-PTSD in both mothers/birthing parents and partners would be beneficial. Early identification of those affected would enable timely intervention and the provision of appropriate treatment options, ultimately mitigating or preventing long-term consequences for affected parents and their families 133 134 .

The WHO recommends improving the subjective birth experience globally, addressing the importance of implementing person‑centered care and avoiding non‑medically necessary and non‑evidence‑based interventions during childbirth 135 . This is consistent with Germany’s national health objective “Health Around Childbirth”, emphasizing the necessity of encouraging mothers/birthing parents involvement and empowerment throughout childbirth 136 . Future research should evaluate the effectiveness of training programs focused on enhancing person‑centered approaches, trauma‑informed care, and cultural competency in obstetric health care settings 85 .

Furthermore, intervention studies should assess the efficacy of supportive care practices that aim to enhance subjective childbirth experiences and, thus, reduce the risk of developing CB-PTSD symptoms. Such research will create valuable evidence to developing best practice guidelines for improving mental health outcomes of mothers/birthing parents, partners and children, ultimately informing clinical and policy approaches to obstetric health care 137 .

### Conclusion

This study highlights the associations between mistreatment and discrimination during childbirth, the subjective birth experience, and CB-PTSD symptoms. In both mothers/birthing parents and partners mistreatment and discrimination during childbirth predicted a more negative subjective birth experience, which in turn contributed to more severe CB-PTSD symptoms. However, among partners, the associations between mistreatment and discrimination during childbirth and CB-PTSD symptoms were only statistically significant in the unadjusted models.

These findings underscore the importance of ensuring respectful, person-centered care during childbirth to prevent both, experiences of mistreatment and negative subjective birth experiences, thereby mitigating the risk of psychological impairments in families. Practical implications include integrating CB-PTSD screenings into routine postpartum care for both mothers/birthing parents and partners, especially in parents with a difficult birth, alongside the implementation of training programs for obstetric health care providers aimed at reducing mistreatment and discrimination during childbirth while enhancing care quality. Further research is crucial to fully understand the potential psychological impact of mistreatment and discrimination during childbirth on both parents, including the underlying physical and psychological mechanisms, with a particular focus on the long-term psychological effects on health-related outcomes, such as CB-PTSD symptoms and their development over time.

## Ethics Statement

The study involving human participants was reviewed and approved by the Ethics Committee of the Technische Universität Dresden (No: SR-EK-331072022).

## Supplementary Material

- Supplementary Table **S1** : Results of attrition analyses.
- Supplementary Table **S2** : Correlations between mistreatment index, discrimination experience, subjective birth experience, CB-PTSD symptoms, and potential confounders for mothers/birthing parents.
- Supplementary Table **S3** : Correlations between mistreatment index, discrimination experience, subjective birth experience, CB-PTSD symptoms, and potential confounders for partners.

## Supplementary Material

Supplementary Material Zusatzmaterial
