# Supplementary material for: Mistreatment and Discrimination during Childbirth, Associations with Symptoms of Childbirth-Related Posttraumatic Stress Disorder and the Mediating Role of the Subjective Birth Experience: A Quantitative Analysis Within the Prospective Cohort Study RESPECT PARENTS
Source: Geburtshilfe Frauenheilkd. 2025 Nov 11;85(12):1304–25. doi: 10.1055/a-2717-7798 (PMC12674902; doi:10.1055/a-2717-7798)
Supplement: Supplementary file 1 — Supplementary Material [file 10-1055-a-2717-7798_27187892.pdf]

Table S1. Results of attrition analyses.

	Mothers/birthing parents				Partners			
	**Competers (*n* = 1091)**		**Non-competers (*n* = 323)**		**Competers (*n* = 465)**		**Non-competers (*n* = 136)**	
**Baseline characteristics**	***M (SD)***	**Range**	***M (SD)***	**Range**	***M (SD)***	**Range**	***M (SD)***	**Range**
Parental age (in years)	**32.17 (4.94)**	**18-45**	**31.06 (5.80)**	**18-46**	34.26 (5.40)	20-54	33.94 (7.06)	19-57
	**t(1410) = -3.39, p < 0.001, Cohen's d = -0.22**							
SES index	**16.22 (2.97)**	**6.5-21**	**14.61 (3.56)**	**4.0-21**	**16.67 (2.75)**	**6.4-21**	**15.12 (3.37)**	**5.8-20.5**
	**t(1314) = -7.21, p < 0.001, Cohen's d = -0.52**				**t(536) = -4.44, p < 0.001, Cohen's d = -0.54**			
Fear of childbirth (W-DEQ, version A score at T1)	65.04 (20.26)	10-134	66.72 (21.09)	6-143	**58.86 (17.42)**	**6-101**	**53.29 (18.58)**	**18-91**
					**t(428) = -2.25, p = 0.025, Cohen's d = -0.31**			
Depressive symptoms (EPDS score at T1)	**6.87 (4.71)**	**0-27**	**8.06 (5.08)**	**0-23**	4.04 (3.61)	0-17	4.80 (4.54)	0-24
	**t(1218) = 3.33, p = < 0.001, Cohen's d = 0.25**							
Anxiety symptoms (GAD-7 score at T1)	4.91 (3.61)	0-21	5.37 (4.17)	0-21	3.78 (3.41)	0-18	4.17 (3.47)	0-19
CB-PTSD symptoms (City BiTS score at T1)	8.58 (9.43)	0-60	9.45 (11.01)	0-56	3.85 (5.96)	0-37	6.56 (9.69)	0-33
	***n*[a]**	**%[b]**	***n*[a]**	**%[b]**	***n*[a]**	**%[b]**	***n*[a]**	**%[b]**
Relationship status								
In a permanent relationship	1.017	98.0	245	98.0	404	99.5	81	100
Not in a permanent relationship	21	2.0	5	2.0	2	0.5	0	0
Mother tongue								
German [c]	993	91.0	222	89.5	424	91.2	71	86.6
Other	98	9.0	26	10.5	41	8.8	11	13.4
First-time parent								
Yes	**619**	**57.0**	**116**	**48.3**	316	68.0	57	72.7
No	**467**	**43.0**	**124**	**51.7**	149	32.0	22	27.8
	**Fisher's exact test: p = 0.018**							

Note. City BiTS = City Birth Trauma Scale; EPDS = Edinburgh Postnatal Depression Scale; GAD-7 = Generalized Anxiety Disorder Scale 7; SES = Socioeconomic status; W-DEQ = Wijma Delivery Expectancy/Experience Questionnaire; T1 = during pregnancy (24th week of gestation onwards); T2 = around eight weeks after the expected birth date.
[a] n varies slightly due to missing data of some participants; [b] valid percent; [c] Including German only and German plus another language.

Table S2. Correlations between mistreatment index, discrimination experience, subjective birth experience, CB-PTSD symptoms, and potential confounders for mothers/birthing parents.

	1	2	3	4	5	6	7	8	9	10	11	12	13	14	15	16	17	18
**1. Mistreatment Index**	1																	
**2. Discrimination experience**	0.32**	1																
**3. Subjective birth experience**	0.30**	0.17**	1															
**4. CB-PTSD symptoms**	0.27**	0.18**	0.46**	1														
**5.** Age	-0.05	-0.01	0.04	-0.04	1													
**6. First-time parent**	0.12**	0.09*	0.12**	**0.11****	-0.27**	1												
**7. SES index**	0.03	-0.02	0.06	**0.07***	0.20**	0.07*	1											
**8. Fear of childbirth**	0.13**	0.11**	0.45**	**0.28****	0.12**	0.03	0.05	1										
**9. Depressive symptoms during pregnancy**	0.13**	0.10**	0.33**	**0.40****	-0.09**	-0.06	-0.14**	0.42**	1									
**10. Anxiety symptoms during pregnancy**	0.14**	0.07*	0.32**	**0.41****	-0.06	-0.10	-0.05	0.40**	0.71**	1								
**11.** CB-PTSD symptoms during pregnancy	-0.05	-0.05	0.01	0.04	0.27**	-0.75**	-0.02	0.14**	0.20**	0.23**	1							
**12. Pregnancy complication**	0.05	0.06	0.12**	**0.12****	0.11**	-0.05	-0.06	0.08*	0.10**	0.12**	0.03	1						
**13.** Birth Place	-0.08**	-0.04	-0.14*	0.01	-0.03	-0.08**	0.01	-0.14**	-0.01	0.01	0.04	-0.06	1					
**14. Birth complication**	0.19**	0.08*	0.22**	**0.16****	-0.02	0.17**	-0.02	0.05	0.03	0.02	-0.11**	0.06*	-0.09**	1				
**15. Birth mode**	0.08**	0.15**	0.22**	**0.07***	0.13*	0.08**	0.01	0.09*	0.03	0.07*	-0.06	0.14**	-0.11**	-0.08*	1			
**16. PTSD prior to pregnancy**	0.11**	0.06*	0.05	**0.11****	-0.01	-0.06	-0.09**	0.07*	0.16**	0.18**	0.11**	0.05*	0.01	-0.01	0.01	1		
**17. Depression prior to pregnancy**	0.09**	0.08**	0.05*	**0.15****	0.08**	-0.01	-0.07*	0.09**	0.16**	0.16**	0.08**	0.03	0.01	0.02	0.03	0.25**	1	
**18. Anxiety prior to pregnancy**	0.10**	0.10**	0.05	**0.11****	0.26	-0.06*	-0.09**	0.06	0.17**	0.17**	0.13**	0.05	-0.04	-0.01	0.04	0.31**	0.29**	1

Note. All associations were calculated using Spearman correlation coefficient.
** p < 0.05. ** p < 0.01.*

Table S2. Correlations between mistreatment index, discrimination experience, subjective birth experience, CB-PTSD symptoms, and potential confounders for partners.

	1	2	3	4	5	6	7	8	9	10	11	12	13	14	15	16	17	18
**1. Mistreatment Index**	1																	
**2. Discrimination experience**	0.43**	1																
**3. Subjective birth experience**	0.21**	0.20**	1															
**4. CB-PTSD symptoms**	0.18**	0.17**	0.34**	1														
**5. Age**	-0.02	-0.02	-0.11*	**-0.12****	1													
**6. First-time parent**	0.09	0.01	0.24**	**0.15***	-0.31**	1												
**7. SES index**	0.06	0.08	0.09*	**0.10***	0.07	0.12*	1											
**8. Fear of childbirth**	0.04	0.07	0.40**	**0.19****	-0.06	0.13*	0.04	1										
**9. Depressive symptoms during pregnancy**	0.10	0.18**	0.32**	**0.42****	-0.08	0.03	-0.14**	0.35**	1									
**10. Anxiety symptoms during pregnancy**	0.11*	0.12*	0.38**	**0.42****	-0.05	0.01	-0.03	0.36**	0.71**	1								
**11.** CB-PTSD symptoms during pregnancy	-0.04	0.05	0.01	0.01	0.15**	-0.61**	-0.05	0.04	0.19**	0.20**	1							
**12.** Pregnancy complication	–	–	–	–	–	–	–	–	–	–	–	–						
13. Birth Place	-0.05	-0.04	-0.10*	0.07	-0.05	-0.05	0.01	-0.06	0.03	0.04	0.05	–	1					
**14.** Birth complication	–	–	–	–	–	–	–	–	–	–	–	–	–	–				
**15. Birth mode**	0.04	0.19**	0.16**	**0.10***	0.05	0.05	0.02	0.04	0.10	0.12*	0.02	–	-0.11	–	1			
**16.** PTSD prior to pregnancy	-0.05	0.03	-0.02	0.08	-0.01	0.06	-0.03	0.04	0.17**	0.18**	-0.02	–	0.03	–	0.04	1		
**17. Depression prior to pregnancy**	0.16**	0.02	0.10*	**0.11***	0.05	0.03	-0.04	0.04	0.18**	0.23**	0.05	–	-0.01	–	-0.05	0.18*	1	
**18.** Anxiety prior to pregnancy	0.01	0.01	0.02	0.07	-0.03	0.04	0.04	0.02	0.14**	0.16**	-0.01	–	-0.05	–	-0.05	0.14**	0.26**	1

Note. All associations were calculated using Spearman correlation coefficient.
* $p < 0.05$. ** $p < 0.01$.
